# Treatment seeking for gambling disorder in nationwide register data – observations around a major shift in legislation

**DOI:** 10.3389/fpubh.2024.1293887

**Published:** 2024-03-19

**Authors:** Anders Håkansson, Anna Karlsson, Carolina Widinghoff

**Affiliations:** ^1^Psychiatry, Department of Clinical Sciences Lund, Faculty of Medicine, Lund University, Lund, Sweden; ^2^Malmö Addiction Center, Malmö, Sweden

**Keywords:** gambling disorder, behavioral addiction, treatment, in-patient, register

## Abstract

**Background:**

Treatment seeking for gambling disorder is known to be low and there has been a lack of longitudinal research regarding treatment opportunities. The present study aimed to assess possible changes in treatment uptake after a formal introduction of gambling disorder in social services and health care legislations, by using register data, including patient characteristics with respect to socio-demographics and comorbidities.

**Methods:**

Nationwide register data were collected for the years 2005–2019, describing diagnoses in specialized out-patient health care and in in-patient hospital care. Numbers and characteristics of patients with gambling disorder were followed longitudinally. Also, a new legislation for treatment by public institutions was introduced in 2018, and data were compared for the years before and after the shift in legislation, both nationally, for each of the three major urban regions, and for the rest of the country. Comparisons were made with respect to concurrent mental health comorbidities, age and gender.

**Results:**

The number of out-patient gambling disorder diagnoses increased over time, but without any significant step changes around the shift in legislation. Over time, patients were younger, became more likely to have gambling disorder as their primary diagnosis, and less likely to have mental health comorbidities, whereas gender distribution did not change. Among the smaller group of patients diagnosed in in-patient settings, mental health comorbidity increased over time. Despite gradual changes over time, no changes in demographics were seen around the actual shift in legislation, although the psychiatric comorbidity appeared to increase after this change.

**Conclusion:**

After the introduction of gambling disorder in the responsibility of social services and health care settings in Sweden, the number of patients diagnosed with gambling disorder increased only modestly. Likely, further implementation of gambling disorder treatment is required in the health care services. Also, longer longitudinal studies are needed in order to understand to what extent patients not seeking health care treatment are received by municipal social services or remain outside the treatment system.

## Introduction

Treatment seeking in patients with gambling disorder (GD) has been reported to be low, and often hindered by a number of barriers both related to the patient and to the organization and availability of treatment (1, 2). Recent meta-analytic data has reported that globally, only around 20 percent of people with a gambling problem seek treatment (3). Thus, although GD is a severe condition typically involving a number of psychosocial and psychiatric consequences (4, 5), many patients appear to remain untreated. Treatment studies have demonstrated mainly an effect of cognitive behavioral therapy, although other psycho-therapeutic and pharmacological strategies also have been tested (6).

Previous research has identified a number of barriers affecting treatment seeking and treatment availability, such as shame, stigma and a preference for any method that allows for an individual to cope with the problem without formal treatment (2, 7). Such barriers to treatment seeking may either be due to the individual’s own psychological and motivational processes, or to other factors in the person’s context (8).

However, organizational factors may also be involved. Few people in the general population may associate GD to the specialized health care or social services settings specialized in addictive behaviors (9).

Being a more recently recognized clinical diagnosis in many settings, GD is likely to have a lower treatment uptake and more organizational treatment barriers than in substance use disorders or in other mental health disorders. In addition, individuals with a GD may be less likely to seek – or to be detected by – specialized health care, than what is seen in other disorders. Hypothetically, this may be due to the non-substance-related nature of the condition, where physical consequences or physical withdrawal symptoms are few, implying that affected individuals might be less likely to precipitate treatment seeking in primary care or in other health care settings.

As in a number of settings, the setting studied here, Sweden, has had markedly under-developed treatment services for GD during decades (10, 11). However, parallel to the emergence of the Internet with new online gambling entities, and the fact that GD was highlighted as an addictive disorder in the DSM-5 in 2013, the academic interest in the field has increased nationally and globally (12). Researchers have called for a systematic reframing of the issues of gambling problems and gambling harm (13), which further underlines the need for global research collaborations and sharing gambling harm prevention strategies internationally. In Sweden, through 2016, it was estimated that less than 1 % of individuals with GD up to then had received a GD diagnosis in specialized health care registers (health care units except for primary care), while its uptake in social services also was believed to be very limited (11). For a long time, treatment and support to GD patients relied upon voluntary peer support groups in a few local settings (14), on a national helpline outside the regular mental health treatment services, and at best on a handful of local initiatives leading to formal treatment services (10). These few treatment services appear to have covered only a small minority of the patients in need for treatment (11). In the meantime, gambling was explicitly not stated in the legislation as one of the addictive disorders which should be treated by municipal social services or in health care, where only alcohol and drug use disorders were formally included. After a number of years of formal treatment in the social services of the major cities in the country, although not based on an obligation in the legislation, the first major specialized health care unit for GD treatment started in one of the urban regions (Skåne) in 2015 (15). From January, 2018, GD was formally entered in the legislation stating the obligations of treating addictive disorders in municipal social services and in the health care services in the entire country (11). This new legislation stated that both the health care services, and the social services, should be able to provide treatment for GD. These organizations are both either public or publicly funded through tax money. While interventions from the social services are provided at no cost to the individual, health care services are paid by the individual only up to an annual maximum amount per year (around 100 Euros), following exactly the same model as for any health care contacts for other mental or physical complaints (a cost that can also – if needed – be covered for the individual by the social services). Patients can voluntarily seek treatment in either social services or in the health care system, or can – from a different health care provider – be voluntarily referred to a health care facility working with addictive disorders such as GD. In a wider perspective, these changes in Swedish legislations intended to improve the process for people with gambling problems to reach professional help, and also provided particular opportunities to study and scientifically describe the shift of a system.

In theory, the formal inclusion of GD in the treatment obligations of major stakeholders in the area was believed to vastly increase the actual treatment uptake for GD. A previous study, documenting specialized Swedish health care contacts under a GD diagnosis through 2016, demonstrated very low treatment uptake in that kind of treatment (11). This theoretically should increase substantially, both as a direct result of the treatment obligation being introduced in 2018 for health care services, but also because an increasing number of patients in the social services likely would lead to a larger number of health care referrals, detection of mental health comorbidities requiring psychiatric consultations, or other collaborations between treatment providers likely leading to increased numbers of patients in both services. It is of importance to understand whether such changes may have taken place, in order to identify treatment gaps and potentially underdeveloped chains of referral for patients with GD in certain regions.

Altogether, it is unclear to which extent the low treatment uptake for GD is only due to individual, psychological factors, and to which extent formal organizational barriers prevent individuals from seeking treatment. Based on this, the present study aimed to observe treatment uptake for GD (ICD-10 code F63.0) in specialized out-patient and in-patient settings in Sweden, year by year, in order to study potential changes over time in age and gender distribution, level of mental health comorbidity and social welfare support, and specifically whether it changed after the formal introduction of treatment responsibility for GD by health institutions in Sweden, on January 1, 2018. In addition, based on the assumingly uneven distribution of treatment historically, changes occurring after the introduction of the legislation in 2018 were studied for the three major urban regions, and for the remaining parts of Sweden, separately. We hypothesized that treatment uptake for GD would increase over time in the entire country, and that a marked increase would be seen after the 2018 shift in legislation. Also, we hypothesized that the proportion of women and patients without mental health comorbidity would increase after the shift in legislation, given the assumption that the increased responsibility of public institutions would broaden the population with GD reached by the health care system, in contrast to the predominantly male population and the high rates of comorbidity seen when treatment uptake was limited in Sweden (11).

## Methods

The present dataset consists of data from the national Swedish patient register (16), a register including all health-care contacts in in-patient hospital settings and in specialized out-patient settings, i.e., in all medical contacts except for primary care (general practitioner) contacts. The register is held by the Swedish Board of Health and Welfare and is commonly used for the observation of treatment uptake in the medical services of Sweden. Data from the Swedish patient register include the diagnoses received at every specialized out-patient or in-patient contact (i.e., all treatment other than out-patient primary care visits). Diagnoses included in the register refer to all primary or secondary diagnoses reported in health care documentation, and do not include information about whether the diagnostic procedures are based on clinical observations or a more structured diagnostic interview or evaluation. In addition, the present study used socio-demographic data derived from Statistics Sweden, which holds register data describing, for example, social welfare support payments or registered unemployment benefits, as well as a long range of other socio-demographic data. Data can be obtained for research after ethics approval and after applications to each of these authorities.

Data included in the present study include in-patient (date of discharge) and out-patient visits from January 1, 2005, through December 31, 2019. The reporting of data related to GD diagnoses has been published before (11), although in a study where treatment uptake and psychiatric comorbidity in GD patients in Sweden was followed only through 2016 (i.e., well before the change in legislation in 2018 which is studied here).

For each of the years (2005 through 2019), unique patients receiving their first GD diagnosis (during the 2005–2019 period) were reported, for out-patient settings and for in-patient settings. This data included only unique patients, i.e., calculating each patient only once. In order to allow the study of a longer post-legislation trend, a sensitivity analysis was carried out, which included the total number of out-patient treatment episodes over time. Here, the total number of contact occasions was recorded, rather than the number of separate patients. This sensitivity analysis allowed for the addition of this type of data for the year of 2020, from this additional data source (a national register study primarily assessing treatment uptake for addictive disorders during the COVID-19 pandemic). The main purpose of this sensitivity analysis was – in addition to the main analysis which covered only two full years post-legislation – to provide a picture of whether a different trend was perceived in the year that followed, i.e., in 2020, the third years after the shift of legislation.

Subjects who were younger than 18 years at their GD diagnosis in the out-patient setting were excluded from the study. This exclusion was done in order to decrease the risk that young individuals with a primary gaming problem were included. In Swedish language, the terms popularly describing a gaming problem are close to the description of a GD diagnosis, and particularly before GD became a well-known and established diagnostic entity in many treatment centers in the health care services, it may be hard to fully distinguish gambling from gaming problems. Gaming disorder treatment has been virtually non-existing in the present setting, but the exclusion of the youngest individuals may have decreased this risk. After excluding individuals with an out-patient GD diagnosis prior to the age of 18, no additional individuals in the out-patient dataset were identified to have received an in-patient diagnosis of GD prior to the age of 18.

The three major urban regions of Sweden, all three with a major urban center, were assessed separately. These include the regions of Stockholm (2.4 million inhabitants), Gothenburg (1.7 million) and Skåne (1.4 million). The Skåne region saw the opening of a specialized, whole-region GD treatment unit in the health care services (within the addiction sub-section of the psychiatry organization) in December, 2015. In Gothenburg, a corresponding whole-region GD treatment unit opened in the health care services in May, 2019. In Stockholm, a specialized clinic is responsible for all addiction treatment in the whole region, and offers (since 2018) treatment for GD in the same treatment context as for alcohol and drug use disorders, at a large number of units throughout the urban area and sub-urban and more rural areas of the region. Thus, no sole, specific GD unit exists in the health care services of the Stockholm region.

Trends over time were calculated for out-patients and in-patients, respectively, and for (1) overall trends during 2005–2019, and (2) for the specific shift around the 2018 legislation, comparing the 2 years after this shift to the 2 years before. Statistical trends over time were analyzed with ANOVA for the continuous variable age, and with chi-square test (linear-by-linear) for the categorical variables (gender, GD as the primary diagnosis or not, and presence of a mental health comorbidity or not). Comparisons of the post-legislation period (2 years), compared to the 2 years preceding the shift in legislation, were carried out on all 1,726 individuals included from these 2 years (972 individuals post-legislation and 754 pre-legislation), using a chi-square test for categorical variables and with a t-test for age. Likewise, the annual percentage of new patients among all patients diagnosed was compared for the 2 years post-legislation compared to the 2 years prior to the legislation. All calculations were made both for the whole country, and for each of the three major urban regions and for the remaining parts of Sweden, respectively. In addition, time series analyses were run, using the annual number of patients or treatment episodes as the outcome variable, with year as the time variable, and comparing the phases prior to, and after, the 2018 shift in legislation, in order to assess potential changes over time and step changes around the shift in legislation, respectively.

The study was approved by the Swedish Ethical Review Authority (file number 2019–01559). In the sensitivity analysis of this paper, adding out-patient data for one additional year from a separate data source, data were derived from a project aiming to follow treatment uptake for addictive disorders during the COVID-19 pandemic, a project that was also approved by the Swedish Ethical Review Authority (file number 2020–04805). No informed consent was required by the ethics authority to be obtained for the present research.

## Results

### Out-patient treatment for GD – overall trends 2005–2019

An out-patient, first occasion episode of GD was seen for a total of 3,186 individuals, including all regions during the whole study period. The numbers of patients, year by year, are displayed in Table 1. Seventy-eight percent were men (*n* = 2,483) and 22 percent were women (n = 703). Mean age at first out-patient GD diagnosis was 35.6 years (std dev 11.1), and median age 34 years (inter-quartile range 27–43). In 56 percent of cases (*n* = 1,777), the GD diagnosis was the primary diagnosis, and in 59 percent (*n* = 1,867) of all cases, there was a concurrent mental health disorder (primary or secondary diagnosis) alongside the GD.

**Table 1:** Out-patient treatment uptake for gambling disorder (GD).

	Skåne (*n*)	Stockholm (*n*)	Gothenburg (*n*)	Other regions (*n*)	Total (*N*)	Mean age (years), all**
2005	23	*	14	50	87	39.5
2006	11	26	18	37	92	39.1
2007	7	22	19	40	88	35.6
2008	15	23	15	40	93	34.8
2009	7	34	22	58	121	35.1
2010	14	35	20	42	111	35.9
2011	12	35	12	55	114	36.5
2012	6	35	13	62	116	36.6
2013	14	68	24	76	182	35.9
2014	22	80	19	73	194	36.1
2015	11	111	21	119	262	35.5
2016	55	136	27	106	324	35.0
2017	88	150	36	156	430	35.4
2018	82	285	40	174	581	35.1
2019	62	159	40	130	391	35.0

Number of new GD patients diagnosed per year, in each of the urban regions, in the remaining non-urban regions, and in the whole of Sweden, as well as the mean age (years) for GD patients each year. ^*^Data missing for 2005 in the register data. ^**^p < 0.001.

In an interrupted time series analysis, an effect of time was seen for an overall increase in the number of new patients with GD (*p* = 0.01), whereas no step change was seen related to the shift in legislation (*p* = 0.32).

Over the study period, mean age of new GD patients decreased significantly (*p* < 0.001, ANOVA, test for linearity). The proportion of women did not change over time (*p* = 0.57, chi-square, linear-by-linear). The proportion of GD as a primary diagnosis increased (*p* < 0.001, chi-square, linear-by-linear) over time, and concurrent mental health disorders decreased over time (*p* < 0.001, chi-square, linear-by-linear). Concurrent (same year) or previous unemployment benefits increased over time (*p* < 0.001, chi-square, linear-by-linear), whereas concurrent (same year) or previous social welfare support decreased over time (*p* < 0.001, chi-square, linear-by-linear).

### Out-patient treatment for GD – trends around the shift in legislation

Regarding the proportion of new patients among all diagnosed individuals during a year, comparing the 2 years after the shift in legislation in comparison to the 2 years before, a significant decrease was seen (from 67 to 63 percent, *p* = 0.01).

When comparing the characteristics of patients in the first 2 years post-legislation to the 2 years preceding the legislation, any concurrent mental health disorder was higher (54 percent, *n* = 521) after the 2018 legislation than before (47 percent, *n* = 356, *p* < 0.01, chi-square = 6.93). GD as the primary diagnosis was not significantly different after the legislation (61 percent, *n* = 595) compared to the 2 years before (65 percent, *n* = 492, *p* = 0.09, chi-square = 2.97). Patients after the legislation did not differ with respect to age (35.0 years) compared to before the legislation (35.2 years, *p* = 0.37, *t* = 0.34), and no difference was seen in the percentage of women after (22 percent, *n* = 209) compared to before (25 percent, *n* = 187, *p* = 0.11, chi-square = 2.61) the legislation. Patients were less likely to be unemployed after than the 2 years before the GD diagnosis (34 vs. 40 percent, *p* = 0.02, chi-square = 5.33), but did not differ with respect to having social welfare support (41 vs. 43 percent, *p* = 0.39, chi-square = 0.73).

Characteristics of GD patients, before and after the legislation, are displayed in Table 2, for each of the three major urban regions and in remaining non-urban regions. After the legislation, gender and age distribution did not change significantly in any of the regions. The presence of GD as a primary disorder decreased significantly in non-urban regions. Except for one urban region, clients were more likely to have a concurrent mental health disorder post-legislation.

**Table 2:** Characteristics of out-patient gambling disorder (GD) patients in each urban region and in non-urban regions in Sweden, during the 2 years before and 2 years after the 2018 shift in legislation.

	Skåne (*n* = 287)		Stockholm (*n* = 730)		Gothenburg (*n* = 143)		Non-urban regions (*n* = 566)	
	Pre-legisla-tion	Post-legislat-ion	Pre-legisla-tion	Post-legisla-tion	Pre-legisla-tion	Post-legisla-tion	Pre-legisla-tion	Post-legislation
Primary GD	80%	78%	81%	79%	38%	26%	46%	37%*
Concurrent mental health disorder	41%	53%*	28%	32%	71%	86%*	65%	76%**
Female gender	24%	27%	21%	18%	30%	26%	28%	22%
Mean age (mean)	34.9	36.2	34.6	34.8	36.1	35.9	35.8	34.7

^*^p < 0.05, ^**^p < 0.01.

### Out-patient treatment for GD – sensitivity analysis

**Figure 1:**
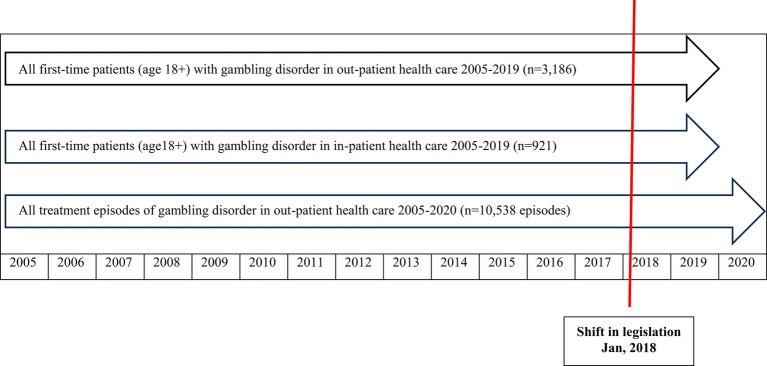
Flow-chart describing datasets and time periods studied, with respect to treatment-seeking for gambling disorder (GD) in Swedish specialized health care 2005–2020, before and after a shift in legislation for treatment responsibility for GD.

In a sensitivity analysis, studying the total treatment uptake described by the total numbers of contacts over years (and including also the year of 2020), there was no indication of a substantial increase in treatment uptake in 2020, and instead rather a decrease. However, in an interrupted time series analysis, an effect of time was seen for overall increase in treatment occasions (*p* < 0.001), and with a step change related to the 2018 shift in legislation (*p* = 0.02). Regarding the number of treatment occasions with GD as the main diagnosis, an effect of time was seen for an overall increase (*p* < 0.01), whereas only a marginally significant increase was seen for episodes with main diagnosis around the 2018 shift in legislation (*p* = 0.05). The total treatment uptake is displayed in Supplementary Figure S1, and the gender distribution in these total treatment contacts is displayed in Supplementary Figure S2 (see Figure 1).

### In-patient treatment for GD – overall trends 2005–2019

Numbers of included patients, year by year, are displayed in Table 3. In addition, for descriptive purposes, numbers are also displayed for each region (Skåne, Stockholm, Gothenburg, and all other regions). Among the total of 923 separate individuals, two were excluded because they were below 18 years of age both in the in-patient and in the out-patient datasets. In an interrupted time series analysis, an effect of time was seen for an overall increase (*p* < 0.01), whereas no step change was seen related to the shift in legislation (*p* = 0.13). Among the remaining 921 individuals, 76 percent were men (*n* = 701) and 24 percent (*n* = 220) were women. Mean age was 38.0 years (std dev 12.5) The proportion of women did not change over time (*p* = 0.70, chi-square, linear-by-linear), and age also did not change over time (*p* = 0.20, ANOVA linear-by-linear). The proportion of first-time in-patient episodes with GD as the primary diagnosis (23 percent, *n* = 215) decreased significantly over time (*p* < 0.001, linear-by-linear), and the proportion of episodes with an additional psychiatric diagnosis other than GD increased over time (*p* < 0.01, linear-by-linear).

**Table 3:** In-patient treatment uptake for gambling disorder (GD).

	Total (*N*)	Skåne (*n*)	Stockholm (*n*)	Gothenburg (*n*)	Other regions (*n*)	GD primary, *n* (%)	Comorbid disorder, *n* (%)
2005	54	4	24	12	14	23 (42.6%)	38 (70.4%)
2006	40	1	17	6	16	15 (37.5%)	28 (70.0%)
2007	45	4	13	8	20	14 (31.1%)	34 (75.6%)
2008	43	3	12	6	22	11 (25.6%)	39 (90.7%)
2009	48	2	11	5	30	9 (18.8%)	40 (83.3%)
2010	51	4	9	16	22	15 (29.4%)	45 (88.2%)
2011	59	6	12	9	32	16 (27.1%)	51 (86.4%)
2012	44	1	8	13	22	10 (22.7%)	41 (93.2%)
2013	53	3	4	8	38	11 (20.8%)	52 (98.1%)
2014	73	6	16	16	35	20 (27.4%)	60 (82.2%)
2015	66	6	8	8	44	9 (13.6%)	62 (93.9%)
2016	61	6	6	11	38	14 (23.0%)	53 (86.9%)
2017	88	9	8	11	60	16 (18.2%)	77 (87.5%)
2018	115	17	17	20	61	19 (16.5%)	99 (86.1%)
2019	81	9	11	16	45	13 (16.0%)	70 (86.4%)
Total	921	81	176	165	499	215 (23.3%)	789 (85.7%)

Numbers per year of new GD patients in each of the urban region and in other, non-urban regions, and in the whole of Sweden. Numbers (*n*), as well as the percentages (*n*, %) of patients each year who have their GD diagnosis as their primary diagnosis, and who have any comorbid psychiatric disorder.

### In-patient treatment for GD – trends around the shift in legislation

In the 2 years post-legislation, compared to the 2 years before, the percentage of GD as the primary diagnosis did not change significantly (from 20 to 16 percent, *p* = 0.36, chi-square 0.83), and nor did the percentage of concurrent mental health comorbidity (from 87 to 86 percent, *p* = 0.78, chi-square = 0.08), the proportion of women (from 24 to 22 percent, *p* = 0.63, chi-square = 0.24), or the mean age (from 37.6 to 36.2 years, *p* = 0.28, *t* = 1.09).

## Discussion

The present study aimed to present possible changes in treatment seeking for GD, and additional related factors, in Sweden, during a period when a major legislative shift took place in order to reach more patients with GD. When including full, nationwide register data of individuals receiving a GD diagnosis in specialized health care between 2005 and 2019, however, only a modest increase in treatment uptake for GD was demonstrated, and the proportion of new patients among all patients seen was modest and decreased slightly around the shift in legislation. While some characteristics of patients changed over the full time period studied, with somewhat younger patients, no significant changes in gender or age were seen across the actual shift in legislation. While psychiatric comorbidity had gradually decreased over time, it became somewhat more likely around the new legislation. More extensive efforts appear to be needed in order to achieve a more substantial increase in treatment uptake for GD in the health care system.

The most recent years, including this new legislation, appear to have seen a new group of patients somewhat more likely to have a concurrent mental health comorbidity. Further studies are required to confirm these findings, but they may indicate that this group, which has historically been difficult to reach (1), has started to receive GD diagnoses and treatment to a higher extent. Also, patients in unemployment were somewhat fewer after the shift in legislation aimed to increase treatment, whereas the proportion of clients with social support was not changed. It can be discussed whether this reflects the fact that people outside of the labor market may remain in the social services and do not receive a GD diagnosis within the health care system.

Psychiatric comorbidity was high, as expected from previous studies (17, 18). Also, as previously described from the present setting, during the period when treatment uptake in this setting was very limited, most likely patients with severe gambling problems and comorbid mental health issues were overrepresented, in comparison to patients with a lower degree of problems (11). In line with this, rates of comorbidity appear to have decreased over time, although a certain increase occurred around the shift in legislation. One possible interpretation of this is that when treatment was officially not mandatory for public institutions, and thereby very hard to access, those who qualified for a GD diagnosis previously had a particularly severe GD with comorbid mental health problems. However, the formal expansion of treatment in 2018 may have changed that and may attract patients with a higher degree of psychiatric comorbidity. Treatment seeking for GD is limited due to perceived barriers to seek treatment (1), and active screening and diagnostic procedures in other sections of health care can be of value.

The increase in comorbidity around the shift in legislation may appear to be somewhat expected, given the formal introduction of GD as a condition to treat, both in social services and within the specialized health care system, where the latter would be expected to address mental health problems to a larger extent. Thus, when psychiatry opened up to GD patients, comorbidity increased. This was the case for most parts of Sweden. It remains to be studied in other study designs, whether patients received in other types of treatment settings, such as in social services, may in fact have lower rates of concurrent mental health problems.

In the current study, in recent years, a lower proportion of patients had recently or previously been unemployed. This can potentially reflect the assumption that broader treatment accessibility may attract patients with a somewhat lower degree of severity in their problems. However, this may also reflect an increased activity in social services in treatment and other support for patients with gambling problems, as potentially patients with social welfare support were received in those contexts rather than in the health care system. More research will have to shed light on these issues, as social services treatment and other treatment outside of the health care services are poorly described in the Swedish setting so far.

The proportion of women among GD patients did not increase over time, in contrast to what has been expected from general population surveys of gambling patterns in women and men (19–21). Considerable differences exist between male and female gambling patterns (22), although a trend over time is that intense gambling patterns and gambling problems in women may become more common, and thereby closer to the male gambling patterns (23). In the most recent survey conducted by the Public Health Agency of Sweden, problem gambling appeared to have increased in women, and in the subgroup of respondents with the most severe gambling problems, the proportion of women was at least as large as the group of men (24). Online gambling has been suspected to recruit women to intense gambling patterns, as they have appeared to constitute a relative majority of problem gamblers in active online gamblers (21), including a particularly high proportion of women in treatment-seeking online casino gamblers (15). Thus, the lack of an increase in the proportion of female patients in the present study is somewhat surprising. Here, more research may be needed. Researchers have called for studies in the area of treatment barriers in women with gambling disorder, where such barriers may differ from those seen in men (25). More research should highlight whether treatment access is evenly distributed between women and men, if diverse barriers to treatment exist between the genders, or if women have increasingly tended to seek treatment in other institutions that the health care system, such as in social services.

The shift in age distribution over time is interesting; over time, patients were gradually younger, although this change was not specifically seen around the shift in treatment legislation, as the change in age distribution appears to have started very early during the study period, with the highest annual mean age data occurring during the very first years of the study. It is possible that this trend may reflect a general shift in problem gambling towards younger individuals, possibly as part of a normalization in the young, as suggested elsewhere (26). Also, the increase of online gambling in recent decades may attract younger individuals than land-based gambling types do (27), which may have contributed to the decrease in age in treatment-seeking patients in the 2000’s. As conclusions are difficult to draw based on the present findings, the age distribution of treatment seeking patients over time, and treatment barriers in different age groups, need to be further analyzed in future studies.

Altogether, over years, even after the shift in legislation making public GD treatment mandatory to provide, the increase in treatment uptake must be described as modest. Also, the present data indicate that treatment in health care even did not continue to increase after the very first year of a new legislation in 2018. Also, the supplementary data added to this study, in order to further shed light on the continuation of treatment access in Sweden after the legislation, demonstrated that treatment in the health care system did not seem to increase in 2020. These findings strengthen a previous picture of treatment barriers in GD, and that policy makers and treatment providers may need to focus specifically on efforts making treatment more available, and trying to overcome inherent psychological barriers against help seeking, such as shame, denial or stigma (2, 7, 8). This also highlights the need for other efforts than only formally making treatment available, such as interventions which address these psychological factors or which emphasize active screening and active referral procedures to treatment (28).

There may be reason to discuss whether the modest increase in treatment uptake, even compared to a period when the treatment of GD was not even mandatory to public institutions, may be due to an overall decrease in the prevalence or severity of GD in the population. However, nothing indicates that this is the case; prevalence data of gambling and problem gambling in household surveys by the Swedish Public Health Agency point to a somewhat increased prevalence of the most severe degree of gambling problems, likely to correspond to a diagnostic level, and in particular among women (24). Likewise, the online gambling market in Sweden remains strong in recent years, including a persistently strong position of rapid, chance-based online gambling on the Swedish gambling market through 2021 (29, 30). Thus, the total number of GD diagnoses in the country is surprisingly low, given the high level of activity in the online gambling market. Altogether, the number of patients diagnosed with a GD annually remained very low in other regions of Sweden than the three most urban regions. Several of the remaining regions have larger urban centers, university hospitals and other major, specialized clinics in psychiatry and addiction, and therefore, the total number of GD patients must be considered insufficient. Here, resources and focused initiatives in this area appear to be needed. Treatment seeking may have increased in social services of the municipality, a treatment provider which is not covered by national registers and from which no data is yet systematically available. While this may seem to be a plausible explanation, it would still be expected that even if patients receive formal treatment in social services, the appearance of mental health symptoms in that setting, or other needs requiring collaboration between disciplines, would also lead to health care contacts. It is difficult to judge to which extent patients are likely to seek social services, clinical psychiatry or both, when they need treatment for gambling problems. In a previous general population survey in Sweden, half of respondents reported that they primarily thought of voluntary peer support organizations as a first contact for GD, and among the remaining respondents, a large majority primarily thought of a health care institution, and only a few percent reported the social services to be the institution to which they would primarily refer a friend or family member with a gambling problem (9). Thus, how problem gamblers act when seeking treatment – for the addictive disorders itself and for concurrent mental health symptoms – is difficult to predict, and likely requires more research with more in-depth research designs than can be obtained from national register data.

Treatment uptake may need to expand in the primary care setting, as well as in specialized psychiatry and addiction psychiatry. Previous researchers in this field have pointed to the importance of general practitioners to address gambling as one of the addictive conditions to screen for and refer for treatment (31), and also that treatment seeking in primary care, such as in Finland, may be too low (32). Primary care is not covered by the present type of patient register in Sweden, but an increased attention to gambling in primary care settings is likely to lead to an increased number of referrals also to specialized psychiatry, and may therefore be fundamental in an improved treatment uptake. More studies of this, including the mapping of how problem gamblers present and are diagnosed in general practitioners’ facilities, are needed, in the present setting and elsewhere. Likewise, online treatment may prevent some factors hindering actual treatment uptake (33). Although this is beyond the scope of the present study, future research should observe whether online treatment options may attract GD patients who have previously abstained from seeking treatment.

In the in-patient data of the present study, comorbid mental health conditions became more common over time. It could be argued that in the relatively rare case of hospitalization of GD patients, hospitals are nowadays more likely to discover gambling problems in patients requiring in-patient care, whereas the gambling problem may have remained undetected before. However, this increase in comorbidity mostly occurred early during the study period, several years before the treatment legislation was even prepared, and did not occur around the actual shift in legislation. Thus, patterns of in-patient treatment episodes for GD over time are difficult to explain. Here, it also should be borne in mind that in-patient GD diagnoses likely reflect episodes of severe mental health complications including suicidal behavior, or a co-detected gambling problem along with a different addictive or other mental health condition requiring in-patient care. Therefore, such episodes are unlikely to include episodes of in-patient care for a behavioral addiction where out-patient interventions are otherwise the most likely intervention. Altogether, diagnoses in the in-patient setting may even be seen as a poor marker of GD treatment uptake in the country, as in-patient episodes are not likely to be driven only by the gambling problem in itself, but rather by gambling-related consequences such as suicidal behavior, or because the gambling problem for some reason has been detected during an in-patient episode primarily initiated because of something else.

Limitations of the present study include the fact that national, register data cannot provide any more detailed information on patients’ gambling patterns or other more individual characteristics. As the diagnoses reported are derived from the national registers, diagnostics cannot be expected to follow the same systematic procedures across settings and individual physicians. Therefore, data can describe change over time only on a macro level. Also, again, health care data do not provide the full picture of interventions for gambling problems, as many attempts to seek help and treatment may happen in other settings, ranging from formal treatment in social services outside the medical area, to brief online or helpline contacts or more voluntarily based peer support groups. However, while the access only to health care data may be seen as a limitation, it also provides a ground for future studies of other treatment seeking patterns. Such future studies should involve a longer longitudinal pattern allowing for larger sample sizes and more strongly statistically powered comparisons across regions and sub-groups in the population. Likewise, regional comparisons are likely affected by a number of co-factors, such as socio-demographic differences, migration background and level of education, which need to be assessed in future studies comparing GD treatment uptake in different regions.

Another limitation is the fact that the time period assessed after the change in legislation is still relatively short, and longer future studies will be of value for increased understanding of possible trends. Here, the supplementary dataset which added a parallel data source for 2020, compared to previous years, prolonged the observational period and confirmed the picture of a very modest treatment increase even after the legislation obliged regions and municipalities to provide GD treatment, and even a further decrease in the third year post-legislation. Further studies should provide a longer follow-up period for GD availability after and before the shift in legislation.

Regarding the expansion of data through 2020, it can be argued that treatment uptake may potentially be limited by the COVID-19 pandemic, and that 2020 is therefore difficult to compare to previous years. While this is a limitation of this analysis, it should not be exaggerated, as the structural changes in mental health treatment availability in Sweden were minor, in the absence of lock-down procedures or home confinement (34). For example, drug prescriptions for mental health disorders were virtually unaltered (35), and an analysis from one region demonstrated that treatment seeking at a gambling disorder unit during 2020 did not change during that year’s early phases of the pandemic (36).

In conclusion, even after a distinct change in legislation, changing public treatment institutions’ previously non-existing responsibility for GD treatment to a full treatment responsibility, the treatment uptake for GD in the specialized health care system remained modest. The present study is one of very few describing the amount and characteristics of treatment seeking patients before and after such a specific intervention attempting to increase treatment availability for GD. Conclusions from the present study are that a widening of treatment access appears to increase the proportion of diagnosed patients who have a comorbid mental health conditions, and over time appears to attract somewhat younger patients, although not in association with the legislation but long before it, and without any obvious change in gender distribution over time. Further efforts are needed in order to increase health care treatment uptake for GD, particularly in regions where focused gambling treatment units are not available, given the severity of GD and its complications. Educational interventions in public health care providers, increased outreach efforts, and expansion of GD treatment to primary care, can be relevant measures in order to increase treatment uptake. Efforts favoring treatment seeking should, however, also be coupled with preventive interventions aiming to postpone gambling onset in the young, and to prevent the migration from low-risk to high-risk gambling modalities. Future studies are necessary in order to evaluate interventions and treatment, and expand the studied groups also to primary care and social services.

## Data availability statement

The original contributions presented in the study are included in the article/Supplementary material, further inquiries can be directed to the corresponding author.

## Ethics statement

The studies involving humans were approved by Swedish Ethics Review Authority. The studies were conducted in accordance with the local legislation and institutional requirements. The ethics committee/institutional review board waived the requirement of written informed consent for participation from the participants or the participants' legal guardians/next of kin because the study included anonymous national registry data.

## Author contributions

AH: Conceptualization, Data curation, Formal analysis, Investigation, Methodology, Project administration, Resources, Software, Visualization, Writing – original draft. AK: Conceptualization, Data curation, Investigation, Methodology, Supervision, Validation, Writing – review & editing. CW: Conceptualization, Investigation, Software, Supervision, Validation, Writing – review & editing.

## Supplementary material

Supplementary Figure S1 Treatment uptake for gambling disorder (GD) during 2005–2020. Total numbers of out-patient treatment contacts (not unique individual patients).

Supplementary Figure S2 Gender distribution in treatment uptake for gambling disorder (GD) during 2005–2020. Percent of total numbers of out-patient treatment contacts (not unique individual patients) representing female patients.
