# Supplementary material for: Treatment seeking for gambling disorder in nationwide register data – observations around a major shift in legislation
Source: Front Public Health. 2024 Mar 19;12:1293887. doi: 10.3389/fpubh.2024.1293887 (PMC10985188; doi:10.3389/fpubh.2024.1293887)
Supplement: Supplementary Figure S1 — Treatment uptake for gambling disorder (GD) during 2005–2020. Total numbers of out-patient treatment contacts (not unique individual patients). [file Supplementary_figures_1_and_2.docx]

**Supplementary figure 1. Treatment uptake for gambling disorder (GD) during 2005-2020. Total numbers of out-patient treatment contacts (not unique individual patients).**

*Data derived from a separate dataset, from a separate study.

**Supplementary figure 2. Gender distribution in treatment uptake for gambling disorder (GD) during 2005-2020. Percent of total numbers of out-patient treatment contacts (not unique individual patients) representing female patients.**

*Data derived from a separate dataset, from a separate study.
